# “It’s within your own power”: shared decision-making to support transitions to buprenorphine

**DOI:** 10.1186/s13722-025-00555-0

**Published:** 2025-03-07

**Authors:** Beth E. Williams, Stephen A. Martin, Kim A. Hoffman, Mason D. Andrus, Elona Dellabough-Gormley, Bradley M. Buchheit

**Affiliations:** 1 Department of Medicine, Division of General Internal Medicine & Geriatrics, Section of Addiction Medicine, Oregon Health and Science University, 3181 SW Sam Jackson Park Rd, Portland, OR 97239 USA; 2 Department of Family Medicine and Community Health, UMass Chan School of Medicine, 55 N Lake Ave, Worcester, MA 01655 USA; 3 Boulder Care, 111 SW Naito Parkway, Suite 200, Portland, OR 97204 USA; 4 Department of Behavioral Neuroscience, Oregon Health and Science University, 3181 SW Sam Jackson Park Rd, Portland, OR 97239 USA; 5 School of Nursing, Oregon Health and Science University, 3076, 3455 SW US Veterans Hospital Rd, Portland, OR 97239 USA; 6 Department of Family Medicine, Oregon Health and Science University, 3181 SW Sam Jackson Park Rd, Portland, OR 97239 USA

**Keywords:** Opioid use disorder, Buprenorphine, Shared decision-making, Patient-centered care, Substance use disorder

## Abstract

**Introduction:**

Buprenorphine is an effective first-line treatment for opioid use disorder (OUD) that substantially reduces morbidity and mortality. For patients using illicitly-manufactured fentanyl (IMF), however, transitioning to buprenorphine can be challenging. Evidence is lacking for how best to make this transition in the outpatient setting. A shared decision-making (SDM) approach has been found to benefit patients with OUD but has not been studied for buprenorphine initiation. We sought to explore participants’ experiences with a SDM approach to buprenorphine initiation.

**Methods:**

Participants were seeking care at a low barrier, telehealth buprenorphine clinic. Clinicians implemented a standardized SDM approach whereby they offered patients using IMF three options for buprenorphine initiation (traditional, low-dose, and QuickStart). They elicited patient goals and preferences and discussed the pros and cons of each method to come to a shared decision. Patients meeting study criteria were invited to participate in semi-structured qualitative interviews 1–2 weeks after the initial visit. Interviews focused on experiences with the clinical visit, suggestions for enhancing the treatment experience, and patient factors affecting the method they chose. Interviews were coded and analyzed using reflexive thematic analysis.

**Results:**

Twenty participants completed interviews. Participants’ mean age was 33, they were 50% female, predominantly white (16 [80%]), and most had Medicaid insurance (19 [95%]). We identified three important themes. First, participants found SDM acceptable and a positive addition to their OUD treatment. They felt their opinion mattered and reported that SDM gave them important control over their care plan. Second, patient goals, preferences, and past experiences with buprenorphine-associated withdrawal impacted what type of buprenorphine initiation method they chose. Finally, participants had advice for clinicians to improve SDM counseling. Participant recommendations included ensuring patients are informed that withdrawal (or “feeling sick”) can occur with any initiation method, that buprenorphine will eventually “block” fentanyl effects once at a high enough dose, and that clinicians provide specific advice for tapering off fentanyl during a low dose initiation.

**Conclusions:**

For patients with OUD using IMF, shared decision-making is an acceptable approach to buprenorphine initiation in the outpatient setting. It can enhance patient autonomy and lead to an individualized approach to OUD care.

## Background

The increasing dominance of fentanyl in the American drug supply has made initiating and safely titrating buprenorphine to an effective dose more difficult than previous transitions from heroin or prescription opioids. Until recent years, the standard approach to initiating buprenorphine in most outpatient settings involved instructing a patient on the time needed to abstain after their last full agonist use (depending on the opioid they are using), then timing the start of buprenorphine with the onset of mild to moderate withdrawal symptoms [1]. While this approach was generally successful during the initial waves of opioid use disorder (OUD), illicitly-manufactured fentanyl’s (IMF) lipophilicity, large volume of distribution, and residual stores have made it particularly challenging to transition to buprenorphine [2, 3].

Virtually all research on transitions from fentanyl has taken place in controlled settings with established research capabilities and funding. The outpatient setting, despite being the most common setting for transition to buprenorphine, is the least studied. Indeed, current literature describes very few outcomes for outpatients using either a low-dose or high-dose approach to transition [4–6]. Recommendations for outpatients most often derive from hospital, Emergency Department, residential treatment, or other mismatched monitored settings. As an example of this mismatch, though low-dose transitions are often used for hospitalized patients, a recent retrospective study found that outpatient retention with this method was only about 20% at 28 days [6]. 

The paucity of understanding as to whether these transition approaches are helpful or harmful is highly problematic for outpatients and limits the informed advice clinicians can provide. In the aggregate, this lack of knowledge leads to a scaled loss of confidence in buprenorphine as a tenable treatment among individuals with OUD using IMF, with predictably disastrous consequences [7, 8].

In the outpatient setting, patients describe a spectrum of negative outcomes as they attempt transition from IMF to buprenorphine [9]. The most severe, if infrequent, of these difficulties is buprenorphine-precipitated opioid withdrawal (BPOW). Additional negative symptoms—while short of BPOW and sometimes described as opioid withdrawal syndrome (OWS)—can also cause harm to patients, both in real time and in the future [10, 11, 12]. These buprenorphine-associated symptoms can be perceived by patients to outweigh its life-saving, but seemingly unattainable, benefits. Even after buprenorphine is titrated to the FDA-advised maximum dose of 24 mg per day, patients may experience ongoing withdrawal symptoms and cravings which require higher buprenorphine doses, especially during the days to weeks after buprenorphine initiation [4, 13]. Informed by these difficult patient experiences and a robust set of supportive data, the FDA has recently made clear its policy that “daily doses higher than 24 mg per day may be appropriate for some patients [14]. 

Transitioning from IMF to buprenorphine in the outpatient setting is a high-stakes and potentially life-saving process. When there are significant limits or ambiguities to medical knowledge that preclude clear recommendations, an evidence-based approach is to engage in shared decision-making (SDM) [15, 16, 17, 18]. Clinicians should have detailed conversations about this transition and the choices patients have in selecting an approach. However, current literature and guidelines do not yet have practical examples of the informed SDM needed in the outpatient setting [19].

Recognizing this major gap in understanding, we sought to study patient experiences during the clinical encounter when a standardized SDM approach was employed to determine their selection of buprenorphine initiation method in a telehealth addiction clinic.

## Materials and methods

### Study setting

The Harm Reduction and BRidges to Care (HRBR) clinic is a low barrier, exclusively telehealth, on-demand addiction treatment clinic that serves patients throughout the state of Oregon [20, 21]. The clinic offers buprenorphine treatment via same-day access, funding for uninsured patients, and a harm reduction approach whereby patients are not required to participate in counseling or be abstinent from all substance use to receive medication. HRBR functions as a bridge clinic in which patients are started and stabilized on buprenorphine, then transitioned to community clinicians for longer-term buprenorphine continuation.

### Study participants and procedures

Prior to study implementation, HRBR clinicians met and agreed upon a standardized approach to SDM. This approach was consistent with the care they had already been providing but included offering the same three methods of buprenorphine initiation to all patients who were medically eligible. The methods offered were decided based on the best available evidence at the time of the research and included: traditional (start 2-4 mg buprenorphine after a period of abstinence from use), low-dose initiation (gradual up-titration of buprenorphine over 4–7 days), and QuickStart (self-administration of intranasal naloxone with concurrent start of 24 mg buprenorphine) [2, 22, 23, 24]. Per clinic protocol, patients with specific situations— certain comorbid conditions, not having a safe place to sleep and reliable access to a bathroom in case they became ill, being without a support person, or who were pregnant—were not eligible to initiate buprenorphine via Quick Start and therefore were not eligible to participate in the study.

The SDM steps included explaining that there were multiple available options, presenting patients with information about the pros and cons of each method, and eliciting patient preferences to come to a decision [15]. Clinicians implemented a standardized set of questions in new patient visits that asked about past experiences with buprenorphine initiation, barriers and facilitators to past transitions, and experiences with precipitated withdrawal. Clinicains also used standard documentation describing the shared decision-making and informed consent process in the encounter note. The clinic had already developed written information about each method that could be provided to patients after the encounter at providers’ discretion.

Initial visits were 60 min for new patients or 40 min for established patients who had returned to IMF use and wanted to restart buprenorphine. This is the time allotted to all HRBR new patients or those with many months since they were last seen. After the initial treatment visit, the study Principal Investigator (BW) approached prospective participants, explained the study’s purpose, and determined their interest in study participation. To meet eligibility criteria, participants were over age 18, reported current or presumed use of IMF or heroin, and were eligible for all methods of buprenorphine initiation as detailed above.

The study team conducted semi-structured qualitative interviews with participants (*n* = 20). The team sought to conduct interviews within 7–14 days of the initial clinical visit, however the protocol allowed for flexibility of interview timing due to challenges with follow up; 75% [15] of the interviews occurred during this timeframe (range 4–93 days). Participants were provided a $40 gift card after completing the interview. Interviews focused on their experiences with the clinic and staff, changes in quality of life related to SDM, and suggestions they might have for enhancing the treatment experience and clinician counseling.

### Data collection and processing

Following informed consent, interviews were conducted via phone or video by 3 study personnel with training in qualitative interviewing (BW, MA, ED). One interviewer (BW) was also a study clinician and did not perform interviews with participants with whom she had conducted the initial treatment visit. Most interviews were conducted one-on-one, while a subset of the initial interviews was conducted by more than one interviewer in order to validate and consider revisions to the study guide. No formal changes were made as a result of this process. Thirty-three patients were invited to participate and 20 completed an interview. The interviews ranged from 15 to 45 min. Interviews were audiotaped and transcribed verbatim with any identifying information removed. The study PI/lead interviewer reviewed all transcripts for accuracy and provided ongoing feedback to the other interviewers to enhance standardization. Basic demographic data including age, gender, ethnicity, and insurance type were obtained via medical chart review. The study was approved by the Oregon Health and Science University Institutional Review Board (IRB #00025462).

### Data analysis

Data were analyzed using Thematic Analysis [25], with an inductive approach. Two coders (KH and BW) crafted the coding framework, with input from an additional study team member (ST). KH and BW created a code book with code definitions and guidelines on how to apply codes to data segments. Next, they iteratively refined the coding framework by applying the preliminary codebook to a sample of the dataset and using a consensus-based approach to discuss their coding and resolve any discrepancies. This process led to the emergence of new codes, the clarification of existing code definitions, and the consolidation of overlapping codes. Using Dedoose (Version 9.2.5), KH and BW coded the data with this framework. As part of the analytic process, the team synthesized the data from specific codes to broader themes by grouping related codes based on conceptual similarities or relationships between codes. Results are reported following the Standards for Reporting Qualitative Research [26].

## Results

Participants were interviewed between May 2023 and February 2024. Participants’ mean age was 33 (18–48). Participants were 50% female, predominantly white (16 [80%]) and most had Medicaid insurance (19 [95%]).

**Table 1:** Participant characteristics (*N* = 20)

Characteristic	*n* (%)
Gender	
Female Male Other^a^	10 (50%) 8 (40%) 2 (10%)
Age (years)	Mean = 33.1 (SD 8.1)
Race (participants could select more than one)	
White American Indian Alaskan Native Black/African American	16 (80%) 2 (10%) 1 (5%) 1 (5%)
Insurance Type	
Medicaid Commercial	19 (95%) 1 (5%)
Type of transition initially chosen	
Low-dose initiation Traditional initiation QuickStart	17 (85%) 1 (5%) 2 (10%)

^a^Transgender/non-binary

The majority of participants chose to initiate buprenorphine via a low-dose initiation (17 [85%]), two participants chose the QuickStart method (10%), and one chose the traditional method (5%). Of note, two participants talked in their interviews about their experiences from a QuickStart initiation they had undergone prior to the study. Three participants (15%) changed their initiation approach between the initial visit and time of the qualitative interview. Eighteen (90%) participants reported they would recommend the buprenorphine method they selected to others. Fewer than half (9 [45%]) reported their substance use was at their desired level at the time of the qualitative interview.

### Theme 1: regaining of control and individualizing care through information sharing

Participants reflected positively on the SDM conversation with the clinician. They appreciated that the clinician took time to detail multiple methods of initiation and describe the way that buprenorphine and fentanyl worked in the body. As one respondent stated *“It’s not just one way*,* which was really helpful knowing that there are a few different ways you could try to administer the buprenorphine. I like that.”* (Participant 15).

Being included in decisions about their care led participants to feel control over the process of buprenorphine initiation. Participants frequently compared their current care with past treatment attempts where they instead described relinquishing control—for example being required to be at a clinical office during buprenorphine initiation or subjected to care parameters that felt punitive or stigmatizing. As one respondent starting via low-dose initiation commented,

*“It’s been good. In the past, just the [OUD treatment] programs… feel more like being in jail. Having somebody with their eye on you the whole time. Threatening they’re gonna take it away if you slip up or till you feel disappointed or guilty or ashamed by them. That’s not something that helps you get better. That’s not something that helps you quit.”* (Participant 1).

In contrast, participants felt empowered and supported by the SDM approach. As one person stated, “*My doctor*,* she told me*,* ‘do what I feel comfortable doing*,*’ you know*,* and that was so nice”* (Participant 6), and another stated, “*no matter what decision I chose it was just a lot of support*.” (Participant 14) One interviewee who chose low-dose initiation described feeling pleased with the approach as it allowed them to feel in control of the process:

*Just that*,* as a patient*,* it’s within your own control to dose it for however long it takes you to get to whatever level you need to. Losing their own power is a big deal with drug users. Just know that it’s within your own power to do the whole thing is a huge deal.* (Participant 16)

Participants also highlighted the patient-centered aspects of the SDM approach, including being able to ask questions and having an individualized plan of care. They understood that experiences with initiation varied widely; SDM enhanced autonomy and allowed patient and clinician to tailor the initiation approach to individual needs. As one respondent put it:

*I think that when it comes to medication-assisted treatment*,* there’s a lot of doctors who just make big assumptions about their patients’ needs. I think that just the recognition that most people know what they want and what they need and when they need it*,* and that just coming into it with more options and listening to people who are in active use more is the most helpful tool.* (Participant 20)

### Theme 2: multiple factors impact initiation method of choice

#### Sub-theme 2.1: timing and access to street drug supply

Respondents were asked to describe their conversation with the clinician and their reasoning for choosing one method of buprenorphine initiation over another. Some participants made the decision based on when they planned to discontinue IMF use. One participant described the decision as a risk calculation after having run out of fentanyl pills and not wanting to use what he considered to be more dangerous fentanyl powder, *“It was just*,* that [QuickStart] was the only option unless I somehow could find blues.”* (Participant 13)

Another participant who chose QuickStart was caretaking for their daughter and described the urgency they felt to discontinue IMF use immediately.

*[The clinician] gave me three different options. She told me with fentanyl*,* some people continue to just use as they gradually get up to the correct amount [of buprenorphine]*,* and I was like*,* that’s something that I don’t want to do at all. I don’t wanna spend the money on it*,* I don’t want to be around it. I have my daughter; I don’t want anything to do with that… I’ve been here for so long at this point that even though the QuickStart method is the more intense*,* hard way*,* I felt like if I didn’t do it then I felt like I was just never gonna do it.* (Participant 12)

#### Sub-theme 2.2: wanting to avoid withdrawal

Other participants based their decision on the perceived risk of withdrawal with each method. Often, they described needing to avoid being sick due to work or familial responsibilities. As one participant undergoing a low-dose initiation described it, “*I have a very active*,* busy lifestyle with lots of things I do every day. I couldn’t continue to live my life the way I do if I had to deal with the sickness of feeling icky*.” (Participant 4).

Multiple respondents echoed having chosen low-dose initiation to avoid uncomfortable withdrawal symptoms. One participant described experiencing a worsening of neuropathic leg pain when in withdrawal and stated, “*I think everybody has their own symptoms of withdrawal. Whether they can handle it or not is a personal thing. For me*,* I just did not wanna have to deal with that pain at all.*” (Participant 16)

#### Sub-theme 2.3: decisions based on knowledge and past experience

Often participants chose a method based on prior experiences with buprenorphine initiation. These experiences included not being successful with other methods in the past or having successfully initiated buprenorphine and then returned to use of IMF. As one respondent undergoing a low-dose initiation stated, “*What played the most into my decision was just my self-awareness of the way my body has interacted with [buprenorphine] versus has interacted with heroin.*” (Participant 20) Another participant had attempted both a traditional and QuickStart initiation in the past. This time, they reported the clinician “*just gave me all the options and asked me which one I would rather do. I did [low dose] cause*,* hopefully no withdrawal.*” (Participant 9)

Another participant had successfully initiated buprenorphine with QuickStart in the past. They had chosen to restart buprenorphine with low-dose initiation because they did not have access to soak in a bathtub, which they felt was essential for getting through the worst phase of withdrawal with the QuickStart method.

Two participants reported coming to the visit already knowing the initiation method they wanted to try. When asked what informed their decision, one reported having discussed it with friends, and another stated simply, “*I like to be educated. I read a lot.*” (Participant 17)

### Theme 3: participants had feedback for clinicians helping patients initiate buprenorphine

#### Sub-theme 3.1: expectation-setting for withdrawal

We asked participants what advice they had for clinicians when counseling patients on buprenorphine initiation. Multiple participants felt it was important to ensure patients understood that withdrawal can occur with any initiation method. One participant undergoing a low-dose initiation commented, “*I know that a lot of times withdrawals are described as flu-like symptoms*,* and that’s true*,* but it’s a lot more than that. There’s a really severe mental aspect of it. ‘Cause when you have the flu you don’t have this crushing anxiety and depression and hopelessness.*” (Participant 3)

Another respondent reflected that clinicians should counsel patients they should not expect to feel “normal” during low-dose initiation, and instead set the expectation that patients may need to take time out of their regular activities to rest. One participant said he would tell others that “*When you are going through this*,* you are going through withdrawal basically. You’re not supposed to go out in 110-degree weather and walk around. It’s like having the flu. You’re supposed to stay at home and relax and just try to get through that part.”* (Participant 6).

One participant who initiated via QuickStart thought clinicians should underscore the potential for severe withdrawal symptoms with this method: “*When somebody is thinking of precipitated withdrawal*,* and they don’t know any better*,* they need to be explained what your body goes through*,* and how bad it’s gonna feel from 1 to 10. I went from 1 to 10 in about a millisecond.”* (Participant 10).

#### Sub-theme 3.2: guidance on IMF use during low dose initiation

Participants highlighted the need for guidance around IMF use during a low-dose initiation. Multiple participants observed the feeling that buprenorphine “blocked” the effects of fentanyl once titrated to a high enough dose. They thought patients should be aware of this so that they are not surprised when they do not get the same effects from using fentanyl, “*Maybe just telling your patients*,* ‘Hey*,* there’s a good chance that it’s gonna start blocking stuff*,* so don’t go crazy.’”* (Participant 11)

Other respondents reported wishing they had specific guidance for tapering off IMF during low-dose initiation. As one person put it, “*I couldn’t really ask a doctor or a pharmacist*,* when I went to pick up [buprenorphine]*,* how much fentanyl I’m supposed to smoke with it. I don’t even know if that’s something that they are okay with. Cause I’ve only heard it from other people who used.*” (Participant 14)

## Discussion

This study supports an SDM approach to buprenorphine initiation in the outpatient setting. Participants reflected favorably on the SDM conversation with their clinician and reported feeling autonomy over their care. Patients’ decision-making process was guided by multiple factors including fear of withdrawal and past experiences with buprenorphine.

Participants in our study described lacking control and feeling excluded from treatment decisions during past episodes of OUD care as has been reported in prior studies [27]. Shared decision-making, defined as “an approach where clinicians and patients share the best available evidence when faced with the task of making decisions, and where patients are supported to consider options to achieve informed preferences” [28] has shown promise for enhancing satisfaction with treatment and improving outcomes in OUD care [16]. This is the first study to examine its application specifically for buprenorphine initiation.

With the current dearth of evidence to guide decision making in the outpatient setting, SDM can provide a useful framework for the buprenorphine initial treatment discussion and could have longer-term implications for patient experience and treatment engagement. For patients with substance use disorders, entry into treatment may be associated with increased stigma; internalization of this stigma has been linked to treatment discontinuation [29, 30]. Past studies have shown that providers who approach patients with OUD with compassion and seek to empower patients can help reduce internalized stigma associated with SUD treatment [30, 31]. Similarly, enhancing patient autonomy and choice during the treatment visit enables patients to reconstruct positive identities in the OUD treatment setting and may influence whether they remain in care [32]. As an approach that seeks to enhance patient control and choice, SDM could help retain patients in care after initiating buprenorphine.

In our study we used a standardized approach to SDM that included offering three buprenorphine initiation methods and eliciting patient preferences and needs to come to a final decision. Future research could work to standardize the implementation of an SDM model for buprenorphine initiation, evaluate fidelity to the model, and measure treatment outcomes [15]. While our study included standardizing parts of history-taking during the initial clinical visit, our findings underscore that eliciting information about social factors affecting timing of initiation, past experiences with initiation and withdrawal, and ultimate goals related to IMF use (i.e., abstinence vs. reduction of use/overdose prevention) are particularly important to include. Development of a graphic SDM tool would also be likely to increase efficacy of the SDM approach [19].

This study identified important themes that guide patient decision-making regarding buprenorphine initiation. The potential experience of withdrawal during initiation was a prominent theme. Participants described planning initiation timing to accommodate feeling “icky,” and choosing an initiation method by weighing withdrawal risk against potential for success. Participants clearly valued expectation setting during the clinical visit as to whether and when to expect withdrawal symptoms. In our study multiple participants changed choice of initiation method between the initial clinic visit and the study interview in accordance with their changing needs, underscoring the dynamic nature of buprenorphine treatment. By applying an SDM approach in follow up visits—including when switching initiation methods or titrating doses—clinicians can ensure that the treatment plan remains in line with patient goals.

The lack of evidence for the frequency of withdrawal and BPOW in the outpatient setting precludes specific counseling on what patients should expect. Withdrawal symptoms have been associated with poorer treatment outcomes, return to illicit use, and increase risk of overdose [3, 22, 33]. One study reported that patients have lower odds of BPOW the longer they wait to initiate buprenorphine after last fentanyl use, but the data did not differentiate odds based on initial starting dose (i.e., low dose vs. standard dose) [12]. A recent retrospective cohort study [9], found that mild withdrawal with low-dose initiation can be common in the outpatient setting, but predictors of withdrawal are still unclear. Prospective studies comparing withdrawal frequency and severity during buprenorphine initiation with use of different methods in the outpatient setting would provide important information to enhance the SDM process.

### Limitations

This study has limitations in generalizability. Its findings may not apply to other locations or settings as the study was conducted at a single clinic providing virtual addiction care in a state with high rates of fentanyl use. In addition, individuals who agreed to participate in the study may have had different opinions or experiences than those who did not volunteer to participate, also limiting generalizability of the results. Clinical visits in this study were not observed or evaluated for conformity to the SDM model. This may limit validity but also enhances the generalizability of findings given that clinical providers vary in their approach in any real-world clinical setting. As the majority of our participants identified as white, opinions and experiences of other racial and ethnic groups—including Black, Latine, and American Indian/Alaska Native—with shared decision-making and buprenorphine initiation cannot be adequately assessed. While there is literature on SDM with a range of racial groups [34, 35], future research should focus on these populations.

## Conclusions

Shared decision-making is an appreciated approach to buprenorphine initiation in the outpatient setting for patients with OUD using IMF. It can enhance patient autonomy and lead to an individualized approach to OUD care. Participants based their initiation decisions on timing and their access to street drugs, avoiding withdrawal, knowledge of initiation techniques, and prior experiences with buprenorphine. More research is needed to determine the efficacy of buprenorphine initiation strategies and how best to involve patients in these decisions.

## Data Availability

Data can be made available upon request from the corresponding author.

## Abbreviations

OUD: Opioid use disorder
IMF: Illicitly-manufactured fentanyl
BPOW: Buprenorphine precipitated opioid withdrawal
OWS: Opioid withdrawal syndrome
SDM: Shared decision-making
HRBR: Harm reduction and bridges to care clinic
